# Endovascular repair of aortic dissection and intramural hematoma: indications and serial changes

**DOI:** 10.1186/2193-1801-3-670

**Published:** 2014-11-13

**Authors:** Eijun Sueyoshi, Hironori Onitsuka, Hiroki Nagayama, Ichiro Sakamoto, Masataka Uetani

**Affiliations:** Department of Radiology, Nagasaki University School of Medicine, 1-7-1 Sakamoto, Nagasaki, 852-8501 Japan

**Keywords:** Aortic dissection, Intramural hematoma, Thoracic endovascular aortic repair, Ulcer-like projection

## Abstract

Thoracic aortic dissection (AD) is one of the most common aortic emergencies. It can be fatal if not promptly diagnosed and treated. Intramural hematoma (IMH) of the aorta is recognized as distinct from classic (double-barreled) AD. IMH also frequently leads to aortic emergency, which can be fatal unless rapidly diagnosed and treated.

Recently, thoracic endovascular aortic repair (TEVAR) has been used for the treatment of complications caused by AD. TEVAR is also a viable option for the treatment of complicated IHM. In this article, we review the details of TEVAR as treatment options for AD and IMH, including the indications for TEVAR, imaging, and follow-up.

## Introduction

Thoracic aortic diseases are life threatening. In the past, surgical replacement was the only effective treatment, involving operations that are technically complex and associated with high-level morbidity (Garzon et al. 2005). Despite the significant improvement of surgical techniques, anesthetic techniques, and perioperative care, transthoracic aortic surgery markedly reduces the risks of serious complications and mortality (5–15% in elective cases and up to 50% in emergent cases) (Ishida et al. 2004).

Numerous studies have reported the safety and efficacy of thoracic endovascular aortic repair (TEVAR) for various thoracic aortic diseases. The most common thoracic aortic pathologic condition amenable to TEVAR is aortic aneurysm, but aortic dissection (AD) has been indicated for TEVAR. Recently, many reports show that TEVAR has been used for the treatment of complications caused by AD (Garzon et al. 2005; Dake et al. 1999).

Intramural hematoma (IMH) of the aorta is now recognized as distinct from AD (Dake 2004). In IMH, ulcer-like projections (ULPs) are also identifiable during follow-up and may be considered as a new intimal disruption (Kitai et al. 2010). Numerous reports on IMH (Sueyoshi et al. 2002) have suggested that ULPs can progress to aneurysms and classic (double-barreled) AD. The role of TEVAR in the treatment of complicated IMH patients, however, remains controversial.

This article presents TEVAR for complicated AD and IMH and serial CT changes following TEVAR. In this article, we review the details of TEVAR for complicated AD and IMH, including the indications for TEVAR, imaging, and follow-up.

### Endovascular repair strategy for patients with AD or IMH

#### Usefulness of TEVAR for AD

When TEVAR is conducted for AD, aortic stability is promoted by thrombosis of the false lumen and stent-induced expansion of the true lumen, and it mimics the healing process (Garzon et al. 2005). Incomplete closure at the entry site and nonclotting of the false lumen may be predictors of a poor prognosis following TEVAR (Czermak et al. 2000). However, TEVAR may still be of merit because it may prevent the false lumen from enlarging, because a high blood pressure is no longer transmitted from the aorta through a large primary tear in the intima (Dake et al. 1999).

Stent-graft placement over the entry site leads to the resumption of normal blood flow in the true lumen and causes false lumen thrombosis (Garzon et al. 2005; Dake et al. 1999; Vedantham et al. 2003). This can cause visceral ischemia if any major branch vessels are supplied by the false lumen. Additional therapies such as percutaneous balloon fenestration, branch vessel stent placement, or aortic stent placement should be conducted in such cases (Garzon et al. 2005; Vedantham et al. 2003).

#### Potential indication of TEVAR for Stanford type A AD or IMH

The management of acute type A AD or type A IMH is life-saving, emergent open surgery. Graft replacement of the ascending aorta, with or without aortic valve repair or replacement is often involved.

TEVAR indications are very limited for type A AD and IMH: (Garzon et al. 2005) retrograde DeBakey type III AD, and (Ishida et al. 2004) residual AD or IMH with complications after surgery. The two above-mentioned conditions can be treated by TEVAR, and a previous report showed that the outcomes are good (Tang and Dake 2009). However, favorable accumulated evidence for TEVAR is insufficient. Further studies are therefore needed (Tang and Dake 2009).

#### Potential indication of TEVAR for Stanford type B AD

The majority of patients with Stanford type B dissection have received medical therapy (Dake et al. 1999). Those with complications typically undergo emergency surgery (Dake et al. 1999). However, both surgical and medical management (blood pressure and heart rate control) sometimes lead to unfavorable outcomes. Patients who have received medical therapy currently show a mortality rate of about 20% (Dake et al. 1999). The mortality rate in surgical repair cases is about 35%, and above 50% for surgical cases involving end-organ ischemia (Dake et al. 1999).

TEVAR has emerged as a less invasive option with low periprocedural mortality and morbidity, particularly for the elderly with serious concomitant illnesses. The 30-day mortality rates are less than 10%, with mortality typically occurring in only those patients with acute presentation and ischemia of organs. These patients show the highest risk for all types of surgical intervention (Garzon et al. 2005).

Regarding TEVAR for AD, type B AD should be divided into two major groups: complicated and uncomplicated type B ADs. Complicated dissection is noted in about 30% of patients who present with acute type B aortic dissection (Criado 2011). Complicated AD refers to evidence of thoracic aortic rupture (hematoma and so on outside aortic wall), malperfusion (ischemia involving the viscera, kidneys, spinal cord, or lower extremities), or rapid expansion in the distal arch or proximal descending aorta to an overall aortic diameter of 4.5 cm or more. Such findings necessitate intervention, as they immediately threaten patient’s lives or limbs (Criado 2011).

Here, we divided type B AD into 4 types based on the therapeutic strategy: 1) acute (within 2 weeks of the initial event) uncomplicated AD, 2) acute complicated AD, 3) chronic (more than 2 weeks from the initial event) uncomplicated AD, and 4) chronic complicated AD.

Acute complicated and chronic complicated type B ADs can be treated with TEVAR. A previous study reported favorable outcomes (Parsa et al. 2010).

A subset of higher-risk patients may be present with uncomplicated ADs who have the potential to benefit from TEVAR. TEVAR in addition to optimal medical treatment is associated with improved 5-year aorta-specific survival rates and delayed disease progression in uncomplicated AD patients. In the presence of stable type B dissection with suitable anatomy, we should consider preemptive TEVAR to improve the late outcomes (Nienaber et al. 2013). Before scaffolding become widely accepted, asymptomatic patients at risk with a partial false critical lumen diameter, lumen thrombosis, or a large entry tear should be considered for TEVAR (Nienaber et al. 2013).

**Figure 1:**
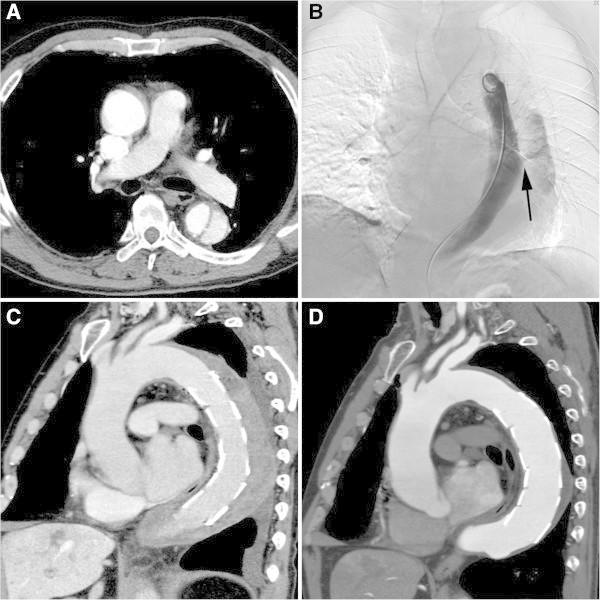
**A 72-year-old male with type B acute AD. A**. One month after onset, contrast-enhanced CT image shows progressive dilatation of the false lumen. **B**. Aortography shows the entry in the descending aorta (arrow). TEVAR (GORE TAG: 34-mm diameter & 20-cm length) was conducted to close the entry. **C**. One week after endovascular treatment, CT image shows thrombosed false lumen. **D**. Two months after endovascular treatment, contrast-enhanced CT image shows that the false lumen has markedly decreased.

**Figure 2:**
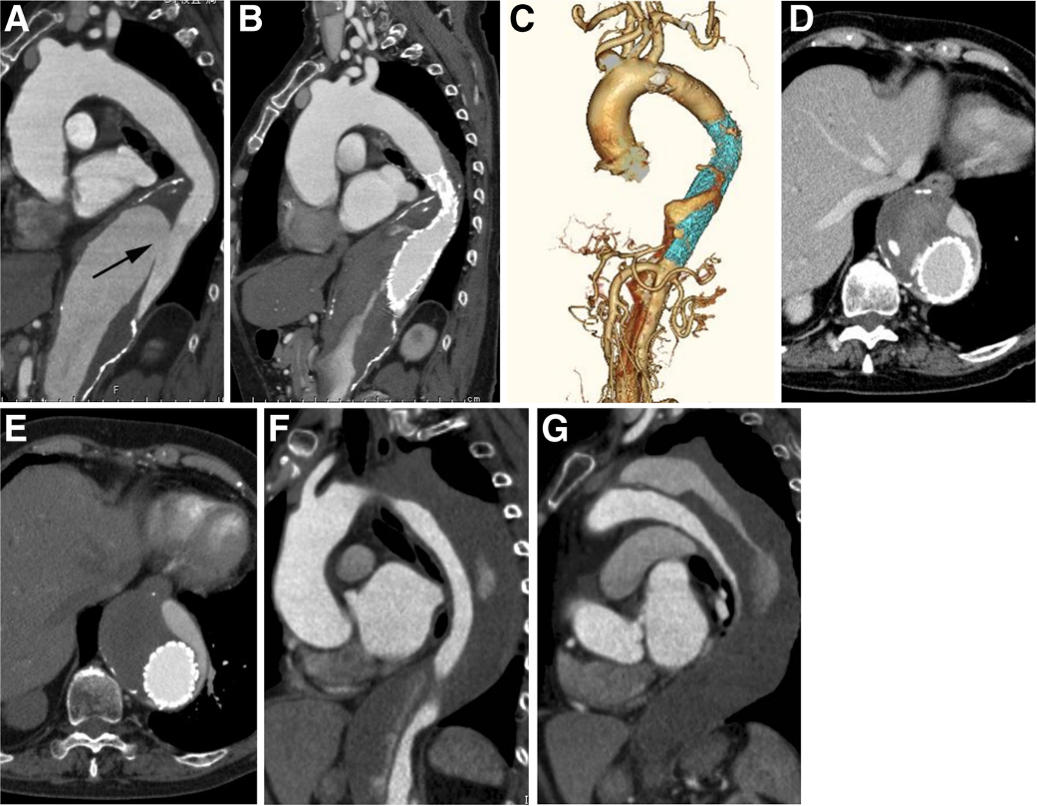
**A 78-year-old female with type B chronic AD. A**. Six years after onset, contrast-enhanced CT image shows aneurysmal dilation of the affected aorta and entry site (arrow). **B**, **C**. TEVAR (GORE TAG: 37-mm diameter & 20-cm length) was performed. Contrast-enhanced CT image and 3D CT angiography show that the entry site was closed. **D**, **E**. Six **(F)** and **(G)** 15 months after TEVAR, contrast-enhanced CT images show that the false lumen has partially thrombosed, but aneurysmal dilation of the affected aorta remains.

Covering the entry site in acute AD may lead to the total regression of the false lumen, and so few additional complete therapeutic procedures may be required (Figure 1). Alternatively, in chronic AD, the false lumen may be partially thrombosed and the intima is more rigid, and so exclusion of the flow into the false lumen causes thrombosis and partial false lumen shrinkage (Garzon et al. 2005) (Figure 2).

### Indications of TEVAR for type B IMH

IMH-related mortality in patients with involvement of the ascending aorta has been considered to be similar to that of AD. Early surgical repair is standard treatment for such patients (Dake 2004). Asymptomatic patients with descending aorta involvement can be monitored closely while undergoing treatment, with intervention only for patients who developing complications such as persistent pain, complicated ULP, signs of impending rupture, progressive maximal aortic wall thickness and compression of the true lumen, an enlarging aorta, or the compromising of a major arterial branch (Dake 2004).

Conventional open surgical repair is associated with high level of morbidity and mortality, particularly in patients who generally are not ideal surgical candidates because they are of an advanced age and/or have coexisting diseases. The 30-day mortality rates after open surgery reportedly range from 10 to 50% (Dake 2004). Less invasive strategies involving the endovascular placement of stent-grafts for the IMH covering have recently been examined.

In type B IMH, patients with complications, such as redissection and complicated ULP, may be treated by TEVAR. Recent studies have revealed that TEVAR yields favorable clinical outcomes (Eggebrecht et al. 2009).

ULPs are sites of intimal disruption. Lesions can show progression to both aneurysms and localized AD. In IMH patients, new lesions or complications may subsequently develop, including ULP, classic AD, or rupture. Recent studies showed that even very early stage intimal defects can be detected by MDCT even. ULPs differ from penetrating atherosclerotic ulcers (PAUs), but their differentiation may be impossible in some patients (Sueyoshi et al. 2002).

Reportedly, complicated ULPs include: (Garzon et al. 2005) progressive aneurysmal dilatation (size of aorta with ULP more than 1.5 times normal) and/or (Ishida et al. 2004) ULP rupture. TEVAR is generally reserved for patients typically categorized as poor open repair candidates. TEVAR has been viewed as optimal for complicated ULPs in patients with type B IMH if anatomically applicable, while open surgery has been employed for non-endovascular treatment candidates (Sueyoshi et al. 2014).

The verification of the short-term safety and efficacy of TEVAR has expanded its application to diverse thoracic aortic pathologies; however, no generally accepted therapeutic regimen has been established for complicated ULPs. Further studies are therefore necessary.

### TEVAR for complicated ULP and serial changes of imaging findings after TEVAR

#### Fate of complicated ULP and IMH after TEVAR

In a prior study, TEVAR is a useful treatment for complicated ULPs in patients with IMH. TEVAR also promotes optimal remodeling of the affected aorta. ULP closure by TEVAR is related to hematoma resorption of the affected aorta by IMH. ULP may provide arterial pressure or blood flow to the false lumen. If TEVAR closes the ULP, arterial pressure or blood flow of the false lumen is reduced or stopped. This may facilitate optimal remodeling of the affected aorta (Sueyoshi et al. 2014). TEVAR can be viewed as an effective alternative to open surgery. Long-term follow-up, however, is necessary to investigate the durability of both the device and TEVAR within the thoracic aorta.

#### Complications after TEVAR for AD and IMH

The mid-term outcomes of TEVAR in chronic type B AD patients were presented in several reviews. The technical success rate was 89.9%. Mid-term mortality was 9.2% and survival rates ranged from 59.1 to 100% in studies with a median follow-up of 24 months. A total of 8.1% of patients developed endoleak, (type I predominance).

**Figure 3:**
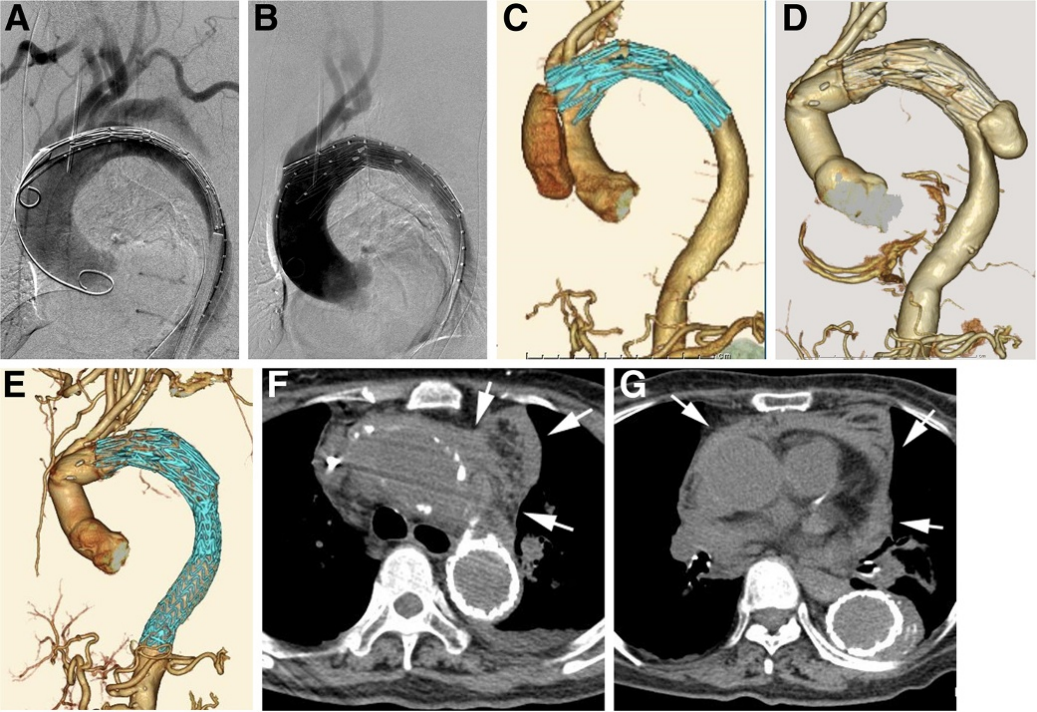
**An 81-year-old female with type B chronic AD. A**-**C**. One year after onset, oblique sagittal MPR images and aortography show aneurysmal dilation of the affected aorta. **D**. TEVAR (handmade stent-graft: 38-mm diameter & 20-cm length) was performed the entry site was closed. **E**. Three months after TEVAR, 3D CT angiography shows a type A AD due to a new intimal tear caused by the proximal edge of the stent-graft. Ascending aorta replacement was conducted. **F**. Three months after surgery, 3D CT angiography shows a type B AD due to a new intimal tear caused by the distal edge of the stent-graft. **G**. Re-TEVAR (GORE TAG: 37-mm diameter & 20-cm length × 2) was performed. 3D CT angiography images show that false lumen fully thrombosed.

Concerning complications after TEVAR for AD, acute major complications included stroke, 3.1%; conversion to type A dissection, 2%; paraplegia, 1.9%; bowel infarction, 0.9%; and major amputation, 0.2% (Figure 3). Aortic rupture was reported at a rate of 0.8% (Thrumurthy et al. 2011).

In the chronic phase, re-intervention rates reportedly ranged from 0 to 60% in studies with a median follow-up of 31 months. Of the patients, 7.8% developed aneurysms of the distal aorta or continued false lumen perfusion with aneurysmal dilatation. Aorto-esophageal fistula, delayed retrograde type A dissection, and neurological complications developed as rare complications (paraplegia, 0.45%; stroke, 1.5%) (Thrumurthy et al. 2011).

Complication rates of TEVAR for complicated ULPs in type B IMH have not been completely unknown. In two of previously reported 18 patients, aortic injury (11%) additionally occurred during follow-up, diagnosed by CT. One patient had retrograde AD 2 years after TEVAR. One patient showed pseudonauerysm development 6 months after TEVAR. Additional endovascular treatment and pseudonauerysm repair were performed (Sueyoshi et al. 2014).

### Stent graft-induced new entry (SINE) tear

Stent graft-induced new entry (SINE) tear developed either perioperatively or during follow-up. The definition of SINE was a new intimal tear caused by the stent graft itself, excluding those resulting from natural disease progression or iatrogenic injury due to endovascular manipulation. In a previous report, proximal and distal SINE represented SINE at proximal and distal ends of the endograft, respectively. Incidence and mortality rates reached 3.4 and 26.1%, respectively.

**Figure 4:**
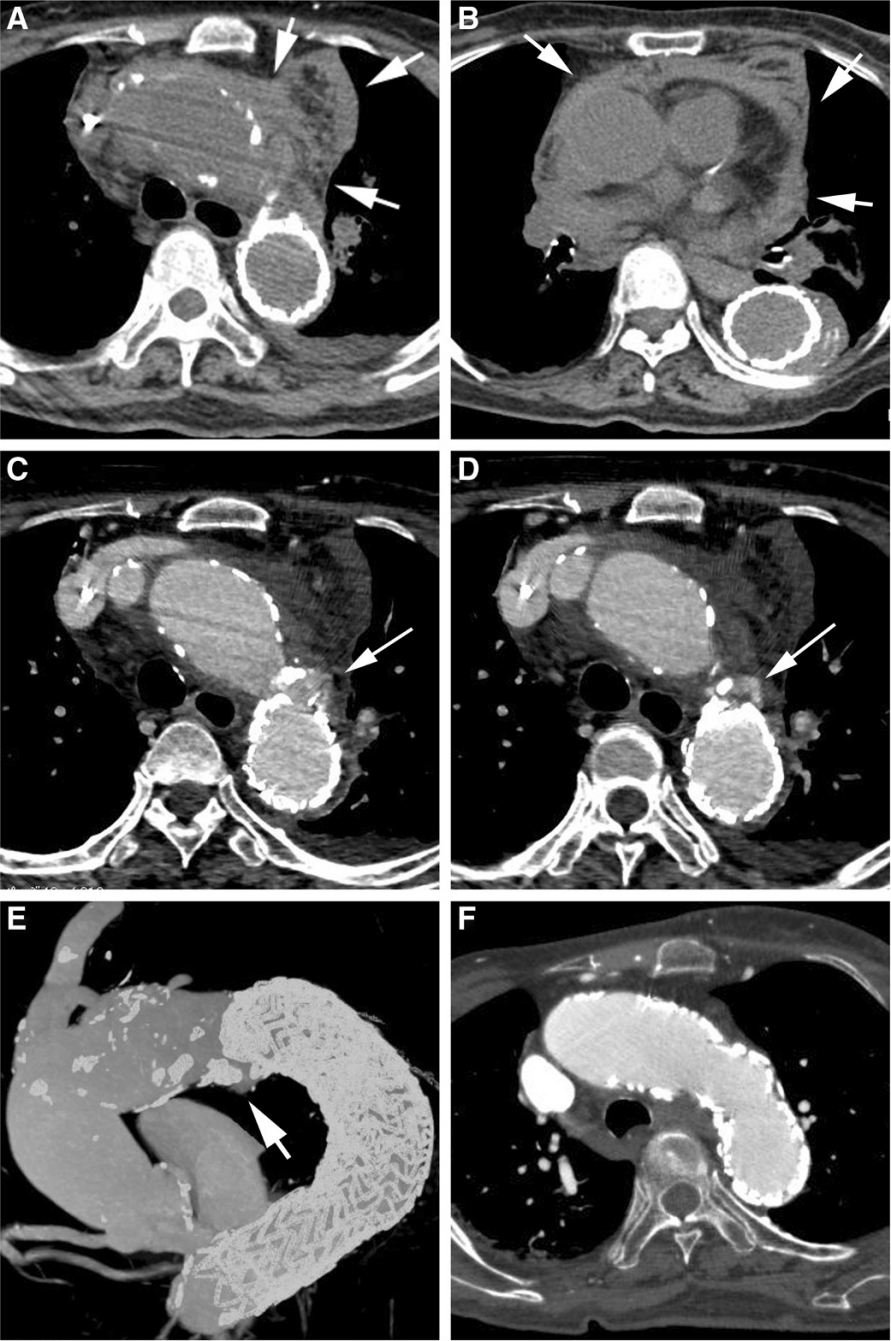
**An 87-year-old female with type B acute intramural hematoma of the aorta (IMH).** One month later form onset, TEVAR (GORE TAG: 37-mm diameter & 20-cm length) was performed because of ULP enlargement. **A**, **B**. One year after TEVAR, this patient complained of severe chest pain. Unenhanced CT images show a mediastinal hematoma (arrow). **C**-**E**. Contrast-enhanced CT and MIP images show that a SINE tear (small pseudoaneurysm: arrows) due to the edge of the stent-graft was noted. Re-TEVAR (GORE TAG: 37-mm diameter & 20-cm length × 2) was performed to close a SINE tear. **F**. Three months after re-TEVAR, a SINE tear and mediastinal hematoma disappeared.

Proximal SINEs are noted at the greater curve of the arch and they cause retrograde type A dissection. The incidence of SINE was 33.3% among Marfan patients vs. 3.26% among non-Marfan patients (P = 0.016) (Dong et al. 2010). Radiologists should be aware that SINE tears can develop in IMH patients as late complications (Figure 4).

## Conclusions

Endovascular techniques have recently been emerging as an alternative treatment for AD or IMH. They constitute a less invasive therapeutic option compared to standard surgery, even when patients have associated diseases that make them poor surgical candidates.

TEVAR’s role in the treatment of AD and IMH remains controversial. However, TEVAR may be a suitable option for the treatment of complicated AD and IHM. TEVAR may contribute to close complicated ULPs and facilitate ideal remodeling of the affected aorta.

Based on our experience, this technique is an effective alternative to open surgery. However, long-term follow-up is required to assess the durability of the device and repair following TEVAR.

### Consent

Written informed consent was obtained from the patient for the publication of this report and any accompanying images.
